# Catalytic Scenarios Over Metal-Carbon Interaction Interface

**DOI:** 10.3389/fchem.2021.810147

**Published:** 2021-12-23

**Authors:** Liwen Xing, Yujuan Jin, Yunxuan Weng, Yongjun Ji

**Affiliations:** ^1^ College of Chemistry and Materials Engineering, Beijing Technology and Business University, Beijing, China; ^2^ Beijing Key Laboratory of Quality Evaluation Technology for Hygiene and Safety of Plastics, Beijing Technology and Business University, Beijing, China; ^3^ School of Light Industry, Beijing Technology and Business University, Beijing, China

**Keywords:** metal-carbon interface, chainmail catalysis, Mott-Schottky effect, single-atom catalysis, carbon-supported nanoparticles

## Abstract

Numerous efforts have been devoted to investigating the catalytic events and disclosing the catalytic nature of the metal-carbon interaction interface. Nevertheless, the local deconstruction of catalytically active metal-carbon interface was still missing. Herein, the selected four types of landmark catalytic paradigms were highlighted, which was expected to clarify their essence and thus simplify the catalytic scenarios of the metal-carbon interface—carbon-supported metal nanoparticles, carbon-confined single-atom sites, chainmail catalysis, and the Mott-Schottky effect. The potential challenges and new opportunities were also proposed in the field. This perspective is believed to give an in-depth understanding of the catalytic nature of the metal-carbon interaction interface and in turn provide rational guidance to the delicate design of novel high-performance carbon-supported metal catalysts.

The tremendous research upon metal-carbon interaction interface has witnessed an extremely rapid development in the field of heterogeneous catalysis, thanks to the progressive advances of synthetic methodologies, characterization techniques, and modeling tools. However, the fundamental understanding of the catalytic nature of the metal-carbon interface has not been profound enough. Up to today, the precise structural description of carbon materials themselves at the atomic level remains extraordinarily difficult owing to the intertwined complexity of crystallinity, doping and surface functionality. What is worse, the local deconstruction of metal-carbon interface was extremely complicated and still ambiguous during the catalytic process, which hinders the deep catalytically mechanistic insights. In this perspective, we first revisited and refined the selected four types of landmark catalytic paradigms and then expected to clarify their essence so as to provide a simplified scope upon the classification of catalytic scenarios of the metal-carbon interaction interface. Finally, the potential challenges and new opportunities were also proposed in the field.

The most common metal-carbon interface should be the carbon-supported metal nanoparticles (NPs) (Figure 1A). For instance, this type of material can be readily obtained by virtue of the adsorption of metal precursors on the selected carbon supports followed by the reduction steps. Since the reactant molecules could have a direct contact with supported metal NPs and were then chemisorbed or activated onto the metallic surface to finish the catalytic reaction, the size, shape, dispersion and exposed facets of large metal NPs on carbon supports largely determined the catalytic activity and selectivity. In such a condition, the role of carbon supports was more likely inclined to anchor or stabilize the metal NPs and prevent their aggregation. Only in some specific cases did both the supported metal NPs and carbon supports behave as a catalyst (Gerber and Serp, 2020).

**FIGURE 1:**
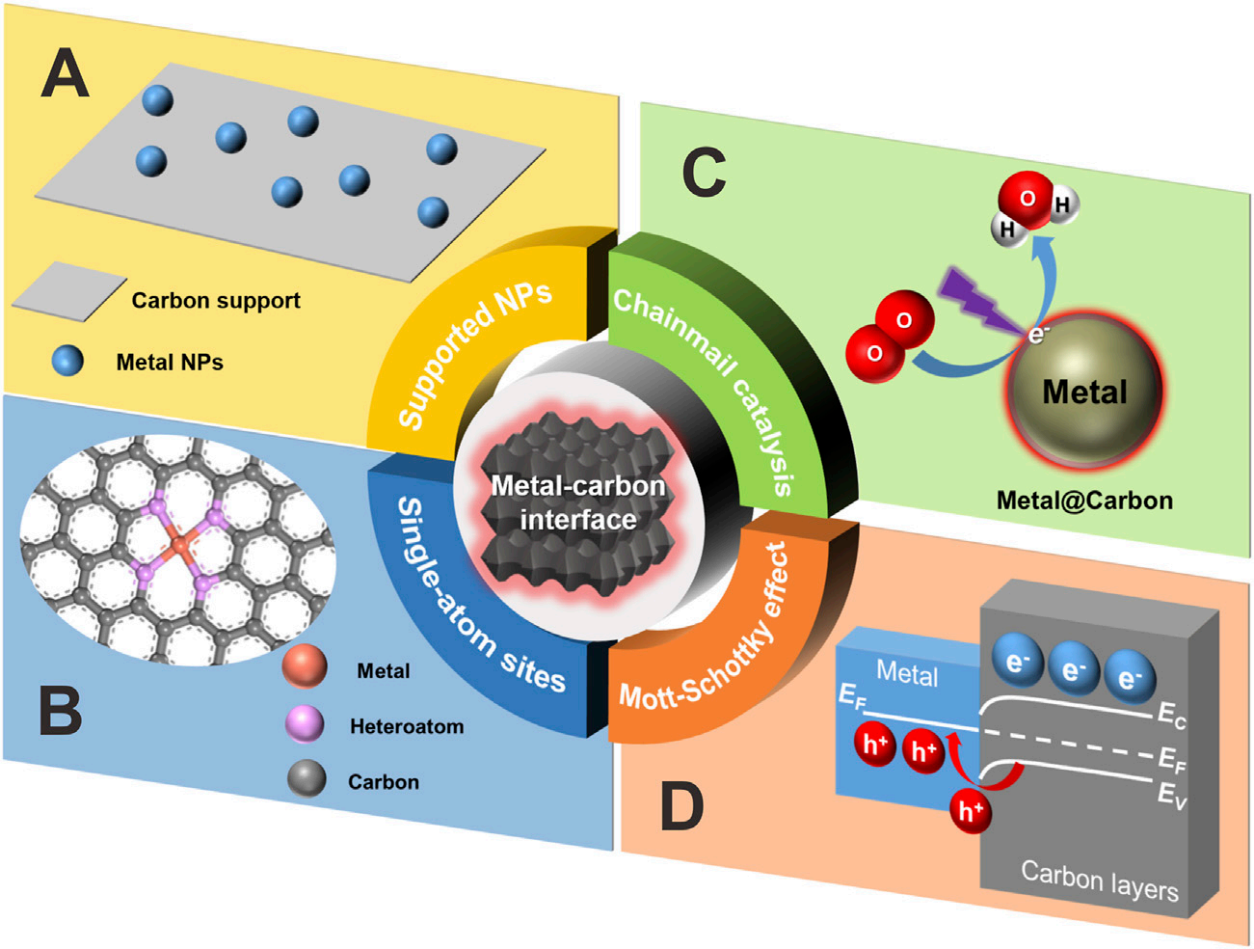
Schematic illustration of the four catalytic scenarios discussed in this perspective. **(A)** Carbon-supported metal nanoparticles. **(B)** Carbon-confined single-atom sites. **(C)** Chainmail catalysis. **(D)** Mott-Schottky effect at the metal-carbon interface. Note that E_C_ and E_V_ stand for energy level of conduction band and valence band, respectively, and E_F_ for Fermi level.

When the size of metal NPs downshifted to the nanocluster (smaller than 2 nm) or even single-atom level, the geometric and electronic structure of metallic sites generated distinct changes compared with those of NPs (Chen et al., 2021), because of the conspicuous exposure of more surface atoms (Liu, 2017). Based on this, the efficiency of atomic utilization could be raised up to nearly 100% and the catalytic activity was expected to be remarkably boosted. Especially for the carbon-confined single-atom metal sites (Figure 1B), the local coordination microenvironment basically conditioned the catalytic selectivity (Li et al., 2020; Sun et al., 2021). The enhanced structural/thermal stability of single-atom catalyst systems could be attributed to the significant metal-support interaction (MSI) (Sun et al., 2020). As for the typical fabrication of carbon-confined single-atom catalyst, the controlled pyrolysis of various metal organic frameworks (MOFs) with thermal stability can be one of the most effective methods.

It has long been traditionally believed that those metal NPs embedded within compact carbon shells were catalytically inactive because of the impermeability of graphitic carbon shells to a gaseous or aqueous reaction environment. In other words, the carbon shells typically impeded the inner metal NPs to directly access the substrate molecules. Afterwards, iron NPs totally encased within pea pod-like carbon tubes (Deng et al., 2013) were prepared by a one-step controlled and reproducible low-temperature thermal treatment of ferrocene and sodium azide followed by dilute acid washing to remove residual metal species outside of carbon walls. The resultant catalyst was found to exhibit electrochemical oxygen reduction activity without obvious decay even in the presence of CN^−^ ions that was frequently used to poison or screen all the active metallic sites exposed to the outer surface. In order to rationalize the abnormal catalytic reactivity, Deng and Bao *et al.* proposed a novel concept called “chain mail for catalysts” (Figure 1C) to describe the possible electron transfer—from the inner metal core to outer substrate molecules—through the intermediate ultrathin carbon shells (Yu et al., 2020). Specifically, 1) The inner metal NPs can form covalent bonds with the adjacent carbon layers by virtue of orbital overlapping, strongly disturbing the localized electron state of both sides. 2) This electron perturbation can be delivered to the external contact area via *π* bonding system of carbon network. 3) Owing to the lower work function of metal NPs, the lost free electrons were transferred to the carbon layer, finally enriching the external carbon surface with electrons. Vividly, the behavior of ultrathin carbon layers might be metaphorically transformed into a chain-mail armor covered on a warrior, which not only offered a crucial body protection from battlefield damages but also guaranteed the full combat capacity of the warrior. Thus, the existence of chain-mail carbon layers instead helped to keep the catalytic reactivity while avoiding the attack from the surrounding harsh environment, which was strikingly different from the conventional wisdom. The catalytic scenario herein actually stressed the significant role of an ultrathin carbon layer in electron transfer events to trigger redox reactions. By virtue of first-principle calculations based on theoretical models, the unique electron-transfer effect of a chain-mail catalyst was further supposed to stand out especially when the thickness of chain-mail layers was less than three graphitic carbon layers. Moreover, a synchrotron-based scanning transmission X-ray microscopy technique was also employed to directly reveal the chemical imaging of the local electronic interaction between chain-mail layers and encapsulated metal NPs (Chen et al., 2015). Since then, the inner enclosed core was also extended to other active species (Zhang X. et al., 2020) such as metal carbide, phosphide etc.

From the solid state physics point of view (Li, 2019), carbon materials, especially those N-doped ones (Zhang J. et al., 2020), might be regarded as a certain type of resin-like polymeric semiconductor due to the tunable composition, bandgap structure and versatile surface chemistry. Thus, Li and Antonietti *et al.*, argued that it was the presence of Mott-Schottky effect between encapsulated metal NPs and adjacent N-doped carbon layers that was really responsible for the catalysis (Li and Antonietti, 2013) (Figure 1D). As a typical synthesis of such a catalyst, it frequently involved the optimized regulation of selected multiple precursors such as metal, carbon, and nitrogen sources, by means of mixing, thermal treatment, and even acid washing. The so-called Mott-Schottky effect above always occurred at the Mott-Schottky heterojunctions where the charges would redistribute until the Fermi level on both sides equilibrated again. In brief, the origin of the driving force to form the local Mott-Schottky heterojunctions lay in the matched difference of the work function between the both contiguous sides. In that way, a charged contact interface was created with electrons accumulated on the carbon layers while the other side (metal-based NPs) depleted in electrons and was thereby positively charged. Namely, a maximal number of electrons would be stored on the N-doped carbon layers until the band bending stopped the current flow and then the catalytic activity basically saturated. As a result, the observed catalytic performance of carbon layers coated metal NPs was strongly dependent upon the Mott-Schottky effect (Su et al., 2017). Especially for photocatalysis, this effect could also prolong the lifetime of charge carriers by enhancing the separation efficiency of electron-hole pairs at the rectifying heterojunctions.

Although their specific apparent forms such as morphology, composition, and structure seemed different at first sight, these four types of catalytic scenarios actually emphasized the unique electronic and geometric effects resulted from active metal centers and/or carbon supports. The primary difference among the four catalytic scenarios might lie in how active metal centers interacted with substrate molecules to accomplish the catalytic loop. According to whether substrate molecules had a direct contact with active metal centers, the four catalytic scenarios discussed in this perspective might be thus categorized into two main classes—direct-contact type (carbon-supported metal nanoparticles and carbon-confined single-atom sites) and indirect carbon layers-mediated electron-transfer type (chainmail catalysis and Mott-Schottky effect). There are still key issues to be addressed in the field of heterogeneous catalysis of the metal-carbon interaction interface.

1) The four catalytic scenarios pointed to a static description of reactive processes while the real catalytic reactions under working conditions might be more inclined to a dynamic one. Indeed, the dynamic and periodic changes (valence state, coordination numbers, and steric configuration etc.) for catalytically active sites were often involved in the realistic catalytic process. Therefore, it was highly desirable to achieve the real-time and real-space high-resolution tracing of those dynamic evolution information, which relied heavily on the innovation of advanced testing tools including *in-situ/operando* characterization techniques.
2) When the catalysts comprised more than one kind of active site, what is the best way to rationally design control experiments to distinguish them from each other as far as the catalytic role was concerned, or to distinguish which one was in the dominant position? For instance, metal-carbon interface derived from MOFs or their composites (Sun et al., 2019) usually involved the coexistence of both carbon-supported/coated metal NPs and single-atom sites, posing a considerable difficulty to the precise identification of intrinsic active sites during catalysis (Lang et al., 2020).
3) Since the fact that the realistic catalytic system can be more complicated than expected, the multiple reactive routes corresponding to different mechanisms would occur and took control of the overall reaction simultaneously (Xing et al., 2020). Here naturally came the question—were there joint schemes of two or more of the catalytic mechanisms described above for a given catalytic system? Except the four catalytic scenarios, were there other new ones in parallel?

In summary, the reasonable solutions to those questions above will not only help to provide further insights into the catalytic nature of the metal-carbon interaction interface, but also in turn provide vital implications for the directional design of high-performance carbon-supported metal catalysts.

## Data Availability

The original contributions presented in the study are included in the article/Supplementary Material, further inquiries can be directed to the corresponding authors.
